# Effects of Esketamine on Emergence Delirium in Children Undergoing Sevoflurane Anesthesia: A Systematic Review, Meta‐Analysis, and Trial Sequential Analysis

**DOI:** 10.1002/pan.70272

**Published:** 2026-08-06

**Authors:** Munder Lateiresh, Abdelaziz Louat, Mukhtar Alwefati, Alhasan Altayf, Matthieu Legrand, Phillip D. Cohen, Eman Abdulwahed, Sapna R. Kudchadkar, Muhammed Elhadi

**Affiliations:** ^1^ Faculty of Medicine Foča University of East Sarajevo Foča Bosnia and Herzegovina; ^2^ Faculty of Medicine and Pharmacy of Casablanca University of Hassan II Casablanca Morocco; ^3^ Faculty of Medicine University of Tripoli Tripoli Libya; ^4^ Faculty of Medicine Azzaytuna University Tarhuna Libya; ^5^ Department of Anesthesia and Perioperative Care University of California San Francisco USA; ^6^ Department of Anesthesiology and Critical Care Medicine Johns Hopkins University School of Medicine Baltimore Maryland USA; ^7^ Faculty of Medical Technology University of Tripoli Tripoli Libya; ^8^ College of Medicine Korea University Seoul South Korea

**Keywords:** anesthesia, emergence delirium, esketamine, meta‐analysis as topic, pediatric patients, preschool, randomized controlled trials as topic, sevoflurane, surgical procedures

## Abstract

**Background:**

Emergence delirium is a frequent and distressing postoperative complication in children, particularly following sevoflurane‐based anesthesia. Despite its clinical importance, the optimal preventive strategy remains under investigation. This study aims to systematically evaluate the efficacy and safety of esketamine in preventing emergence delirium in pediatric patients undergoing sevoflurane‐based general anesthesia.

**Methods:**

We systematically searched seven databases and registries through 27 March 2026. Randomized controlled trials that compared esketamine with placebo in children undergoing surgical procedures were included. The primary outcome was the incidence of emergence delirium. Secondary outcomes were postoperative pain scores, extubation time, post‐anesthesia care unit stay, and postoperative nausea and vomiting. Data were synthesized using random‐effects models, with risk ratios for dichotomous outcomes and mean differences or standardized mean differences for continuous outcomes. Meta‐regression and trial sequential analysis were performed. The risk of bias was assessed using the revised Cochrane risk‐of‐bias tool for randomized trials, and the certainty of the evidence was evaluated using the Grading of Recommendations, Assessment, Development, and Evaluation approach.

**Results:**

Eight randomized controlled trials involving 916 patients were included. Esketamine significantly reduced the incidence of emergence delirium versus placebo (risk ratio = 0.35; 95% confidence interval: 0.26–0.48; *p* < 0.0001). No significant differences were observed between dosing regimens, surgical types, routes of administration, or timing of administration. Pain scores were significantly lower with esketamine (standardized mean difference = −0.58; 95% confidence interval: −0.79 to −0.37; *p* < 0.0001). Extubation time was modestly prolonged (mean difference = 1.72 min; 95% confidence interval: 0.20–3.24; *p* = 0.0267). Meta‐regression revealed a statistically significant association between emergence delirium risk ratios and anesthesia duration (β = 0.98, *p* = 0.042), with no other significant associations observed for the other covariates. Post‐anesthesia care unit stay showed no statistically significant difference, while postoperative nausea and vomiting was significantly reduced with esketamine (risk ratio = 0.69; 95% confidence interval: 0.49–0.97; *p* = 0.0304). No study was at high‐risk of bias; four studies raised some concerns. Certainty of evidence was moderate, except for extubation time, which was low.

**Conclusions:**

Esketamine demonstrates promising efficacy for preventing emergence delirium and improving postoperative pain in children.

**Prospero:**

CRD420251230407.

**Abbreviations**
ASA: American Society of Anesthesiologists
BMI: body mass index
CI: confidence interval
ED: emergence delirium
GRADE: grading of recommendations, assessment, development, and evaluation
IQR: interquartile range
MD: mean difference
NMDA: N‐methyl‐D‐aspartate
PACU: post‐anesthesia care unit
PONV: postoperative nausea and vomiting
RCTs: randomized controlled trials
REML: restricted maximum likelihood
RoB 2: risk of bias 2 tool
RR: risk ratio
SD: standard deviation
SMD: standardized mean difference
TSA: trial sequential analysis

## 1 Introduction

Emergence delirium (ED) is a constellation of cognitive and behavioral disturbances, including agitation, crying, shouting, violent movements, and confusion, that occur during the early phase of recovery from anesthesia [1]. Although ED is generally self‐limited, it can expose pediatric patients to the risk of self‐harm, prolong post‐anesthesia care unit (PACU) stay, increase the need for nursing support, and escalate healthcare costs, and cause significant anxiety and distress for parents and caregivers [2]. ED is regarded as a multifactorial postoperative neurobehavioral disturbance influenced by patient‐related factors (e.g., younger age and preoperative anxiety), anesthetic‐related factors (particularly volatile anesthetics), and surgical factors (e.g., ophthalmologic and otolaryngologic procedures) [3, 4].

Sevoflurane is widely used in pediatric anesthesia due to its rapid induction, rapid emergence, and low airway irritancy [5, 6]. However, sevoflurane anesthesia is a well‐established risk factor for emergence delirium in pediatric patients [7]. This likely reflects the combination of rapid recovery due to low blood solubility and young children's limited capacity to adjust to sudden, unfamiliar surroundings.

Multiple pharmacological interventions have been explored to reduce the incidence of ED, including fentanyl, propofol, midazolam, racemic ketamine, and dexmedetomidine [8]. However, there are risks related to these medications, such as postoperative nausea and vomiting (PONV), delayed recovery, psychotomimetic effects, and inconsistent efficacy [8, 9, 10, 11]. Esketamine, the S‐enantiomer of ketamine, acts as a noncompetitive N‐methyl‐D‐aspartate (NMDA) receptor antagonist and is increasingly used for pediatric premedication due to its superior receptor affinity and anesthetic potency compared to the racemic mixture [12, 13]. Despite its clinical advantages, the use of esketamine requires careful consideration of its dose‐related safety profile and potential psychomimetic effects. However, recent systematic reviews suggest that esketamine may be better tolerated than racemic ketamine [14]. Its administration in pediatric patients is still linked to dose‐dependent adverse events. Specifically, dose‐finding studies in children have identified nausea, vomiting, increased secretions, and neurobehavioral disturbances such as emergence delirium as major adverse events following esketamine administration [15]. Such psychomimetic potential necessitates precise dose titration to ensure a favorable balance between clinical efficacy and the risk of distressing postoperative reactions in the pediatric population. Consequently, several randomized controlled trials have assessed its efficacy in reducing ED in children undergoing sevoflurane‐based general anesthesia [16, 17, 18, 19, 20, 21, 22, 23]. However, their findings remain inconclusive due to small sample sizes, methodological variability, and contradictory conclusions. While previous meta‐analyses, such as Yang et al. [24], have explored this topic, our study offers a more focused contribution by concentrating exclusively on sevoflurane‐based anesthesia—a primary trigger for ED. Furthermore, by employing Trial Sequential Analysis (TSA), meta‐regression, and GRADE assessment, this study provides a comprehensive synthesis of the current evidence to better understand the role of esketamine in this high‐risk population.

## 2 Methods

This systematic review and meta‐analysis was conducted in accordance with the Preferred Reporting Items for Systematic Reviews and Meta‐Analyses (PRISMA) 2020 guidelines, and methodological quality was assessed using the Measurement Tool to Assess Systematic Reviews (AMSTAR) 2 [25, 26]. The corresponding checklists are provided in Tables S1–S3. The study protocol was registered in PROSPERO under the registration number CRD420251230407.

### 2.1 Eligibility Criteria

Studies were eligible if they: (1) were randomized controlled trials (RCTs); (2) compared the effect of esketamine with placebo (saline) on emergence delirium; (3) included pediatric patients < 18 years and classified as American Society of Anesthesiologists (ASA) physical status I or II; (4) used sevoflurane‐based general anesthesia, in which sevoflurane served as the primary maintenance agent for unconsciousness; and (5) involved a surgical procedure (diagnostic procedures, such as fiberoptic bronchoscopy, were excluded to ensure population homogeneity). Studies were excluded if they: (1) failed to meet the inclusion criteria; (2) utilized continuous intravenous infusions of esketamine; (3) focused on long‐term behavioral changes (e.g., negative postoperative behavioral changes) rather than immediate emergence delirium as the primary focus; (4) were observational studies or non–peer‐reviewed publications (e.g., book chapters, editorials, letters, opinion articles, review articles, or animal studies); or (5) contained irretrievable, overlapping, or incomplete data. No restrictions were applied regarding the type of surgery, language, geographical region, or year of publication.

### 2.2 Search Strategy and Study Selection

A comprehensive search was performed on 27 March 2026 across six electronic databases (PubMed, Scopus, Embase, Web of Science, the Cochrane Library, and Google Scholar) and one registry (ClinicalTrials.gov). The search strategy incorporated terms related to “pediatrics,” “esketamine,” and “sevoflurane,” with the three main concepts combined using the “AND” Boolean operator. The full search strategy is presented in Table S4. No filters were applied regarding language, publication year, or study design. Reference lists of included studies, the “cited by” and “similar articles” functions in PubMed, and Google Scholar were manually searched to identify additional records. The screening process comprised two phases: title and abstract screening, followed by full‐text screening. In both steps, two reviewers (M.A., E.A.) independently assessed each article against the predefined eligibility criteria using the Covidence platform [27]. Any discrepancies were resolved by a third reviewer (M.L. or A.L.).

### 2.3 Data Extraction

Data extraction was independently performed by two reviewers (M.A. and E.A.) using a standardized, piloted form, with a third reviewer (M.L.) resolving any disagreements. The extraction form was designed to capture: (1) study characteristics (first author, year, sample size, type of surgery, and Anesthesia details including sevoflurane concentrations); (2) patient baseline variables (age, sex, and clinical settings); and (3) primary and secondary outcome data. To address potential clinical heterogeneity, we systematically extracted data on surgical categories and the use of adjunct analgesia. Outcome data for emergence delirium and PONV were prioritized for collection immediately after extubation, while postoperative pain scores were extracted at the earliest available time point to ensure consistency across the included trials.

### 2.4 Outcomes Definitions

To ensure cross‐trial comparability and clinical relevance, study groups were predefined as subanesthetic (≤ 0.3 mg/kg) or anesthetic (≥ 0.5 mg/kg). The primary outcome was the incidence of emergence delirium during recovery from anesthesia. Secondary outcomes included postoperative pain scores, extubation time, length of PACU stay, and incidence of PONV. Detailed definitions of outcomes across studies are provided in Table S5.

### 2.5 Risk of Bias Assessment

Two reviewers (M.A., E.A.) independently assessed trial quality using the Risk of Bias 2 (RoB 2) tool across five domains: randomization process, deviations from intended interventions, missing outcome data, measurement of the outcome, and selection of the reported result [28]. Each study was rated as low risk, high‐risk, or raising some concerns. Discrepancies between reviewers were resolved through discussion with a third reviewer (M.L. or A.L.). The overall risk‐of‐bias assessments were synthesized and graphically summarized using the Robvis tool [29].

### 2.6 Certainty of Evidence

The certainty of evidence for each outcome was assessed using the Grading of Recommendations, Assessment, Development, and Evaluation (GRADE) framework across five domains: (1) risk of bias, (2) inconsistency, (3) indirectness, (4) imprecision, and (5) publication bias [30]. Evidence could be upgraded in the presence of a large effect, a dose–response gradient, or plausible confounding factors. Based on these assessments, the certainty of evidence was categorized as high, moderate, low, or very low [31, 32, 33, 34, 35, 36, 37, 38]. A summary of findings table was generated using the GRADEpro GDT tool [39].

### 2.7 Data Analysis

Statistical analyses were conducted using R (version 4.4.3) with the “meta” and “metafor” packages [40]. Continuous data reported as medians with interquartile ranges (IQRs) were converted to means and standard deviations using the method of Wan et al. to minimize potential bias [41]. Data were pooled using a random‐effects model with restricted maximum likelihood (REML) estimation to account for both within‐study and between‐study variability. Dichotomous outcomes were expressed as risk ratios (RRs). In contrast, continuous outcomes were reported as mean differences (MDs) or standardized mean differences (SMDs), with 95% confidence intervals (CIs), for outcomes measured on different scales. The magnitude of SMDs was interpreted as small (< 0.2), moderate (0.2–0.8), or large (> 0.8). Heterogeneity was assessed using Cochran's Q test (*p* < 0.10 indicating statistical significance) and quantified using the *I* ^2^ statistic, interpreted as low (< 50%), moderate (50%–75%), or substantial (> 75%). Prediction intervals (PIs) were calculated to estimate the expected range of true effects across different populations [42].

Predefined subgroup analyses for the primary outcome included dose category (subanesthetic ≤ 0.3 mg/kg vs. anesthetic > 0.5 mg/kg) evaluated exclusively for the intravenous route, specific intravenous dosing regimens (0.25 and 0.5 mg/kg), route of administration (intranasal vs. intravenous), timing of administration (before induction, 5–10 min before the end of surgery, or immediately before the end of surgery), and surgical specialty (ophthalmic vs. otolaryngologic surgery) to evaluate the impact of different levels of procedural stimulation. Analyses of secondary outcomes were conducted for the first three subgroup categories to ensure cross‐trial comparability and to identify potential sources of heterogeneity. Leave‐one‐out sensitivity analyses were performed for all outcomes to evaluate the influence of individual studies on pooled effect estimates. Publication bias was assessed through visual inspection of contour‐enhanced funnel plots and Egger's regression test, with *p* < 0.1 considered indicative of potential publication bias [43]. A trim‐and‐fill analysis was also performed to evaluate the potential impact of missing studies on pooled estimates.

Meta‐regression analyses were performed using the study‐level averages of age, body weight, duration of anesthesia, and duration of surgery as covariates for ED risk ratios. For each study, the average was calculated as the mean of the treatment and control group means, reflecting the overall study‐level estimate in the absence of baseline imbalance between groups. Additional meta‐regression analyses were conducted using esketamine dose as a covariate to explore dose–response relationships. Statistical significance was defined as *p* < 0.05. To further reduce the risk of spurious conclusions arising from sparse data, heterogeneity, and repeated testing in cumulative meta‐analysis, TSA was performed. TSA was conducted only for the 0.25 mg/kg and 0.5 mg/kg dose subgroups to avoid double‐counting of the control groups. A retrospective TSA (RTSA) was conducted using the RTSA package (version 0.2.2) in R. The required information size (RIS) was estimated under a random‐effects model with a two‐sided α of 5% and β of 20%, assuming an anticipated relative risk reduction (RRR) of 60% based on the pooled effect from a recent meta‐analysis [44]. Variance inflation was adjusted using the TSA‐corrected τ^2^, and the model allowed for two potentially missing trials to account for small‐study effects. Lan–DeMets α‐ and β‐spending boundaries were applied to determine whether firm evidence was achieved before reaching the RIS. Conclusions are considered definitive if the cumulative sample exceeds the RIS, the Z‐curve crosses the monitoring boundary, or enters the futility zone after surpassing the conventional significance threshold [45].

## 3 Results

### 3.1 Study Selection and Characteristics

From the 2281 articles initially identified, 243 duplicates were removed. After title‐abstract screening, 2010 records were excluded, and 28 full‐text articles were assessed for eligibility. Eight RCTs comprising 976 pediatric patients (916 eligible for analysis) were included. The article selection process was illustrated in the PRISMA flow diagram (Figure 1). The sample sizes of the included studies ranged from 80 to 204 participants. Demographically, the included patients had a mean age of 5.30 ± 2.11 years, a mean weight of 21.07 ± 7.43 kg, and a mean body mass index (BMI) of 17.38 ± 4.46 kg/m^2^. Sevoflurane was used for maintenance anesthesia at 2%–4%.

**FIGURE 1:**
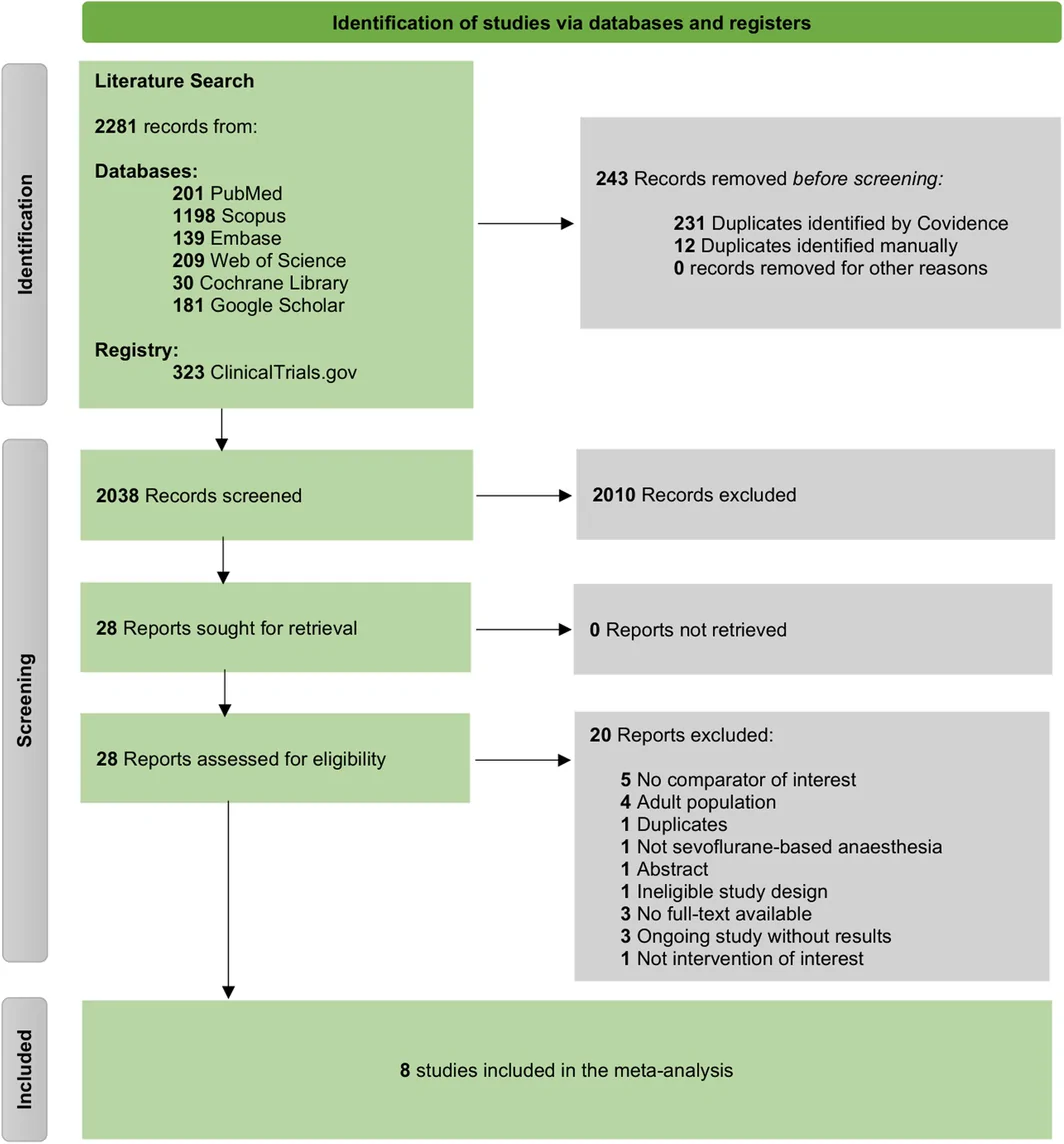
PRISMA Flow Diagram of the Study Selection Process.

The surgical procedures investigated varied, predominantly including strabismus surgery, adenotonsillectomy, and other otolaryngology surgeries. Esketamine was administered via two primary routes: intranasal and intravenous. Dosing regimens were categorized into subanesthetic (≤ 0.3 mg/kg) and anesthetic (≥ 0.5 mg/kg) doses. Specifically, subanesthetic doses included 0.2 mg/kg and 0.25 mg/kg, while anesthetic doses ranged from 0.5 mg/kg to 1.0 mg/kg, with 0.5 mg/kg being the most frequently studied anesthetic dose. Additional baseline characteristics of the included participants are summarized in Table 1.

**TABLE 1:** Characteristics of included studies.

Study ID	Sample size^a^ T (976)	Age range (years)	ASA	Arm +Dose	Route of Administration	Analyzed Patients T (916)	Gender (*n*)		Weight (kg) Mean ± SD	BMI (kg/m^2^) Mean ± SD	Surgical procedures	Anesthesia Sevoflurane induction (%)	Anesthesia Sevoflurane maintenance (%)	Outcome measurement scales
							Male	Female						
Xu 2025 [16]	90	2–5	I‐II	Esketamine 0.5 mg/kg	Intranasal	28	17	11	16.67 ± 2.34	16.2 ± 1.8	Elective strabismus surgery	5%	2%	ED: PAED PACU discharge: Modified Aldrete
				Esketamine 1.0 mg/kg		29	16	13	16.33 ± 3.9	15.8 ± 1.6				
				Saline 0.9% (Control)		28	18	10	15.67 ± 1.56	15.7 ± 1.7				
Liu 2022 [17]	90	3–6	I‐II	Esketamine 0.5 mg/kg	Intranasal	30	—	—	15.77 ± 2.33	—	Elective strabismus surgery	6%	3%–4%	ED: PAED Pain scores: CHEOPS
				Esketamine 1.0 mg/kg		30	—	—	16.47 ± 1.67	—				
				Saline 0.9% (Control)		30	—	—	16.04 ± 1.56	—				
Li 2025 [18]	204	3–6	I‐II	Esketamine 0.5 mg/kg	Intranasal	67	49	18	—	15.7 ± 1.2	Adenotonsillectomy	3%	2%–4%	ED: PAED Pain scores: CHEOPS PACU discharge: Modified Aldrete
				Esketamine 1.0 mg/kg		66	38	28	—	15.5 ± 1.04				
				Saline 29.85% (Control)		67	43	24	—	15.6 ± 1.3				
Wu 2025 [19]	160	2–7	I‐II	Esketamine 0.25 mg/kg	Intravenous	40	26	14	18.0 ± 2.7	15.1 ± 1.5	Otolaryngology surgery	Etomidate 0.3 mg/kg Sufentanil 0.2 𝜇g/kg Cisatracurium 0.1 mg/kg	2%–3%	ED: PAED Pain scores: FLACC
				Esketamine 0.5 mg/kg		40	30	10	18.4 ± 4.3	15.4 ± 2.2				
				Saline 0.9% (Control)		40	30	10	18.1 ± 4.6	15.4 ± 1.8				
Zhaoxia 2022 [20]	80	3–10	I‐II	Esketamine 0.25 mg/kg	Intravenous	20	16	4	24.3 ± 6.4	—	Elective tonsillectomy and/or Adenoidectomy	8%	2%–3%	ED: 4‐point agitation score Pain scores: Modified CHEOPS PACU discharge: Aldrate revised PONV: 4‐point PONV
				Esketamine 0.5 mg/kg		20	13	7	22.4 ± 6.2	—				
				Esketamine 0.75 mg/kg		20	10	10	20.7 ± 6.7	—				
				Saline 0.9% (Control)		20	10	10	24.0 ± 6.4	—				
Chen 2023 [21]	108	3–7	I‐II	Esketamine 0.2 mg/kg	Intravenous	54	33	21	22.3 ± 5.9	15.7 ± 2.6	Tonsillectomy and/or adenoidectomy	8%	2%–3%	ED: Aono Pain scores: CHEOPS PACU discharge: Modified Aldrete
				Saline 0.9% (Control)		54	32	22	22.3 ± 6.0	16.6 ± 3.0				
Li 2022 [22]	80	2–7	I‐II	Esketamine 0.25 mg/kg	Intravenous	40	22	18	17.2 ± 1.9	23.4 ± 3.5	Tonsillectomy	Fentanyl 3 μg/kg Propofol 3 mg/kg Cisatracurium 0.12 mg/kg	2%–4%	ED: RSS
				Saline 0.9% (Control)		40	21	19	16.7 ± 2.1	23.6 ± 3.2				
Jiang 2025 [23]	164	3–10	I‐II	Esketamine 0.1 mg/kg	Intravenous	37	20	17	28.54 ± 7.38	19.05 ± 3.85	Tonsillectomy and adenoidectomy	Fentanyl 3 μg/kg Propofol 2 mg/kg Rocuronium 0.6 mg/kg	3%	ED: PAED/Watcha Pain scores: FLACC PACU discharge: Modified Aldrete PONV: 4‐point PONV
				Esketamine 0.3 mg/kg		39	20	19	29.05 ± 8.53	19.52 ± 6.58				
				Esketamine 0.5 mg/kg		37	26	11	29.80 ± 9.88	19.39 ± 7.23				
				Saline 0.9% (Control)		40	18	22	29.98 ± 9.61	21.41 ± 8.14				

Abbreviations: CHEOPS (scale), Children's Hospital of Eastern Ontario Pain Scale; ED (incidence), Emergence Delirium; FLACC (scale), Face, Legs, Activity, Cry, Consolability; *n*, number; PACU (discharge readiness criteria), Postanesthesia Care Unit; PAED (scale), Pediatric Anesthesia Emergence Delirium; PONV (incidence), Postoperative nausea and vomiting; RSS (scale), Ramsay Sedation Scale; Watcha (scale), Watcha Behavior Scale.

^a^ Sample sizes represent the number of patients randomized in each study.

### 3.2 Risk of Bias Assessment

None of the included studies was judged to be at high‐risk of bias. Four studies raised concerns, primarily related to the randomization process; one study also raised concerns about selective reporting [17, 19, 20, 22]. All trials were assessed as having a low risk of bias for deviations from intended interventions, missing outcome data, and outcome measurement. Overall, four studies were rated as low risk of bias, whereas four were judged to raise some concerns. Detailed risk‐of‐bias assessments are presented in Figures S1 and S2.

### 3.3 Primary Outcome

#### 3.3.1 Emergence Delirium

The ED showed a significant risk reduction in the esketamine group, both with the subanesthetic dose (RR = 0.43, 95% CI [0.25; 0.73], *p* = 0.0016, *I* ^2^ = 58%) and the anesthetic dose (RR = 0.21, 95% CI [0.12; 0.35], *p* < 0.0001, *I* ^2^ = 0%) compared with the placebo group, with no significant differences between the two dose levels, *p* = 0.055 (Figure 2a). The overall pooled analysis also demonstrated a significant reduction in ED incidence in the esketamine group compared with controls (RR = 0.35, 95% CI [0.26; 0.48], *p* < 0.0001), with moderate observed heterogeneity (*p* = 0.0197, *I* ^2^ = 47.0%) (Figure S3).

**FIGURE 2:**
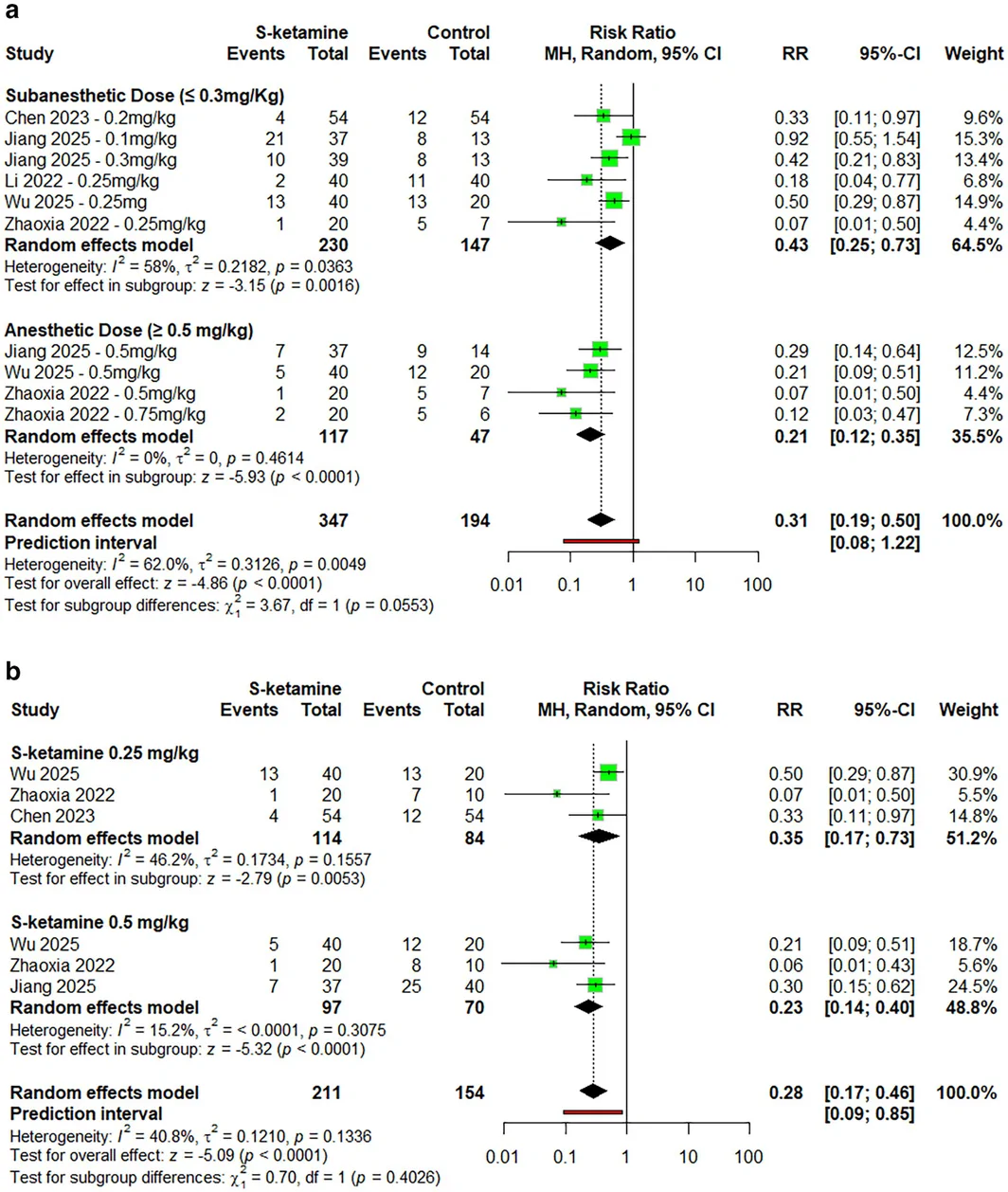
Forest Plots of Emergence Delirium (ED). (a) Subgroup Analysis of Emergence Delirium According to Anesthetic Level (Subanesthetic Vs Anesthetic). (b) Subgroup Analysis of Emergence Delirium According to Specific Esketamine Doses (0.25 and 0.5).

Similarly, significant reductions in ED incidence were observed at 0.25 mg/kg (RR = 0.35, 95% CI [0.17; 0.73], *p* = 0.0053, *I* ^2^ = 46.2%), 0.5 mg/kg (RR = 0.23, 95% CI [0.14; 0.40], *p* < 0.0001, *I* ^2^ = 15.2%), with no significant differences between different dose regimens, *p* = 0.40 (Figure 2b). Both intravenous (RR = 0.30, 95% CI [0.18; 0.50], *p* < 0.0001, *I* ^2^ = 65%) and intranasal (RR = 0.39, 95% CI [0.28; 0.56], *p* < 0.0001, *I* ^2^ = 0%) administration were associated with statistically significant ED risk reduction, with no significant difference between the two routes, *p* = 0.4012 (Figure S4). Further subgroup analysis, based on the timing of administration and surgery type (otolaryngologic vs. ophthalmologic procedures), showed consistent decreases in ED risk, with no significant differences between subgroups (Figures S5 and S6).

#### 3.3.2 Secondary Outcomes

A moderate reduction in pain scores was observed in the esketamine group for both the subanesthetic dose (SMD = −0.48, 95% CI [−0.88; −0.09], *p* = 0.0161, *I* ^2^ = 71.5%) and the anesthetic dose (SMD = −0.97, 95% CI [−1.26; −0.67], *p* < 0.0001, *I* ^2^ = 8.4%) (Figure S7). The overall pooled analysis revealed a moderate reduction in pain scores (SMD = −0.58, 95% CI [−0.79; −0.37], *p* < 0.0001), accompanied by moderate heterogeneity (*p* = 0.0036, *I* ^2^ = 59.0%) (Figure S8). Significant reductions were also found across specific dosing regimens: moderate at 0.25 mg/kg (SMD = −0.78, 95% CI [−1.05; −0.51]; *p* < 0.0001, *I* ^2^ = 0%); and large at 0.5 mg/kg (SMD = −1.04, 95% CI [−1.37; −0.71], *p* < 0.0001, *I* ^2^ = 8.6%) (Figure S9), with no significant differences detected between the dosing subgroups.

Subgroup analysis by route of administration revealed significant pain reductions for both intravenous (SMD = −0.68, 95% CI [−0.97; −0.38], *p* < 0.0001, *I* ^2^ = 69.2%) and intranasal (SMD = −0.41, 95% CI [−0.62; −0.21], *p* < 0.0001, *I* ^2^ = 0%) administrations, with no significant difference between the two routes (*p* = 0.15) (Figure S10).

There was no statistically significant increase in extubation time at the subanesthetic dose (MD = 0.55 min, 95% CI [−0.37; 1.48], *p* = 0.2408, *I* ^2^ = 54.2%), whereas the anesthetic dose was associated with a significant prolongation (MD = 5.72 min, 95% CI [1.21; 10.23], *p* = 0.013, *I* ^2^ = 76.7%), with significant differences between subgroups (*p* = 0.028) (Figure S11). The overall pooled analysis demonstrated a significant increase in extubation time (MD = 1.72 min, 95% CI [0.20; 3.24], *p* = 0.0267), accompanied by substantial heterogeneity (*p* < 0.0001, *I* ^2^ = 78.6%) (Figure S12).

Numerical increases that did not reach statistical significance were also observed at the specific doses of 0.25 and 0.5 mg/kg across both routes of administration, with no statistically significant differences between subgroups (Figures S13 and S14).

No significant effect of esketamine on the length of PACU stay was observed with the subanesthetic dose (MD = −0.08 min, 95% CI [−2.31; 2.16], *p* = 0.947, *I* ^2^ = 70.1%), and the anesthetic dose (MD = 5.22 min, 95% CI [0.03; 10.40]; *p* = 0.048, *I* ^2^ = 88.5%), while the overall pooled analysis (MD = 1.02 min, 95% CI [−0.73; 2.77], *p* = 0.25), with substantial heterogeneity observed (*p* < 0.0001; *I* ^2^ = 81.1%) (Figures S15 and S16). Similarly, no significant effects were detected at specific doses of 0.25 or 0.5 mg/kg, nor with administration routes (Figures S17 and S18). No statistically significant differences were observed between all PACU subgroups.

The incidence of PONV did not differ significantly between the dose subgroups (*p* = 0.240) at either the subanesthetic dose (RR = 0.73, 95% CI [0.49; 1.08], *p* = 0.1163, *I* ^2^ = 0%) or the anesthetic dose (RR = 0.44, 95% CI [0.21; 0.92], *p* = 0.029, *I* ^2^ = 0%), while there was a statistically significant effect of esketamine on PONV in the overall pooled analysis (RR = 0.69, 95% CI [0.49; 0.97], *p* = 0.03, *I* ^2^ = 0%), with no evidence of heterogeneity (Figures S19 and S20). Nonsignificant effects were observed at specific doses of 0.25 mg/kg and 0.5 mg/kg (Figure S21). Nevertheless, no increased risk was detected with intranasal administration (RR = 1.28, 95% CI [0.42; 3.86], *p* = 0.66, *I* ^2^ = 0%), whereas the intravenous route was associated with a statistically significant reduction in PONV (RR = 0.65, 95% CI [0.46; 0.92], *p* = 0.015, *I* ^2^ = 0%) (Figure S22).

A comprehensive summary plot was generated to provide an integrated visual synthesis of effect estimates, 95% confidence intervals, statistical significance, and *I* ^2^ across all evaluated primary and secondary outcomes (Figure 3).

**FIGURE 3:**
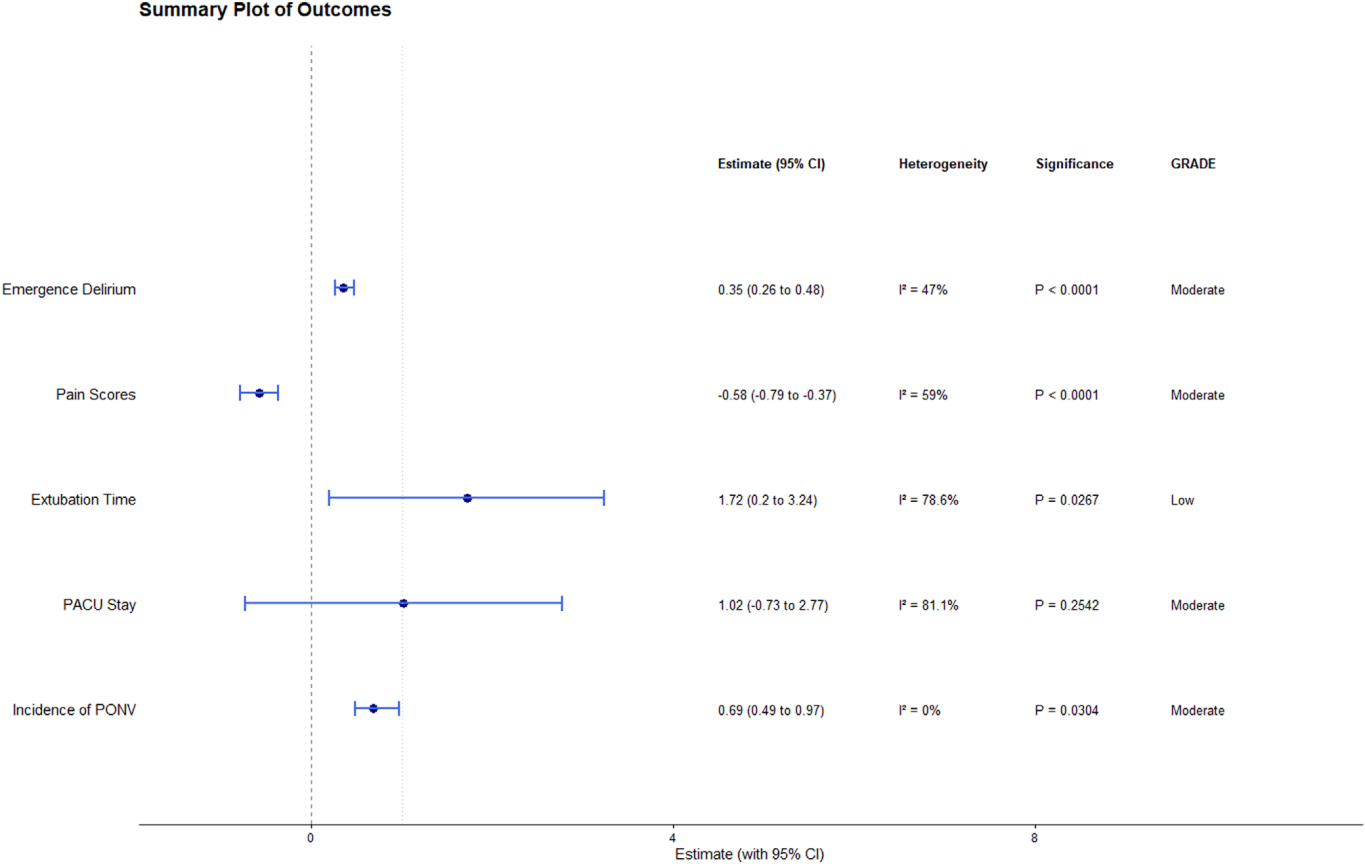
Summary Plot of the primary and secondary outcomes.

### 3.4 Meta‐Regression and Trial Sequential Analysis

Meta‐regression revealed a statistically significant association between ED risk ratios and anesthesia duration (β = 0.98, *p* = 0.042). In contrast, no statistically significant associations were observed with other study‐level covariates, including average age, body weight, esketamine dose, extubation time, or PACU stay. The combined meta‐regression bubble plots are presented in Figure 4a,b.

**FIGURE 4:**
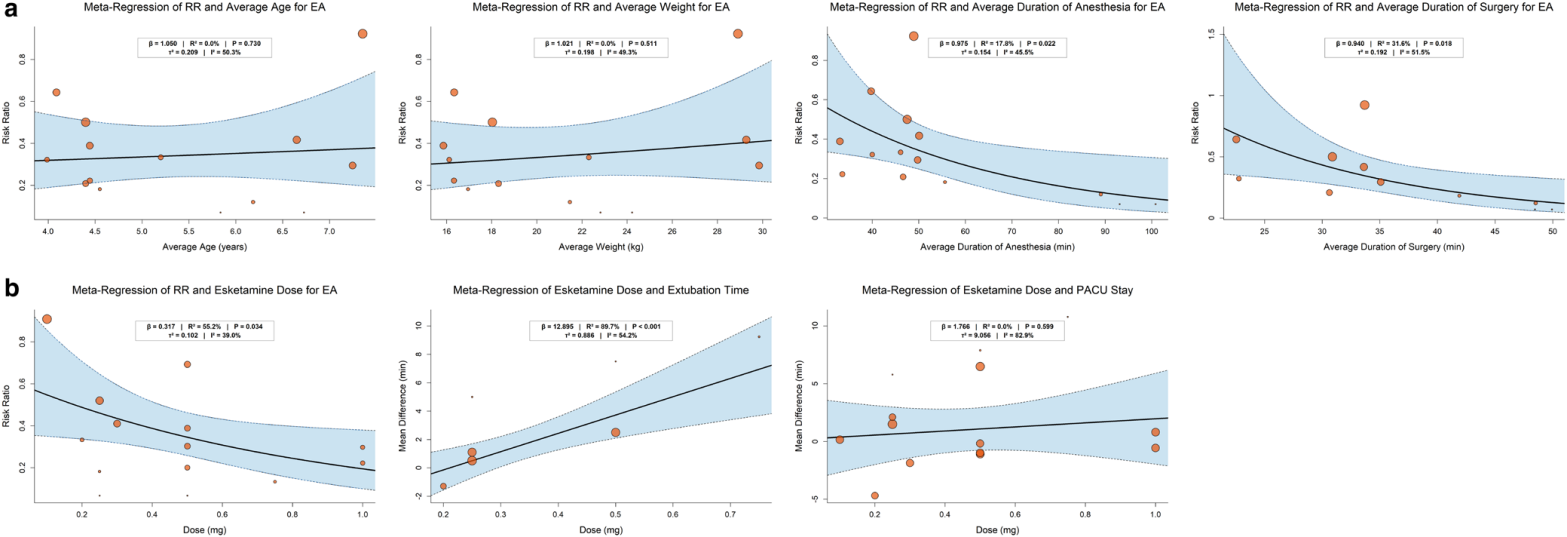
Meta‐Regression Bubble Plots. (a) Meta‐Regression Analysis of Emergence Delirium According to Anesthesia Duration. (b) Meta‐Regression Analysis of Emergence Delirium According to Esketamine Dose.

TSA showed that for both 0.25 and 0.5 mg/kg doses, the cumulative *Z*‐curve crossed the conventional significance and α‐monitoring boundaries, indicating conclusive evidence Figure 5a,b.

**FIGURE 5:**
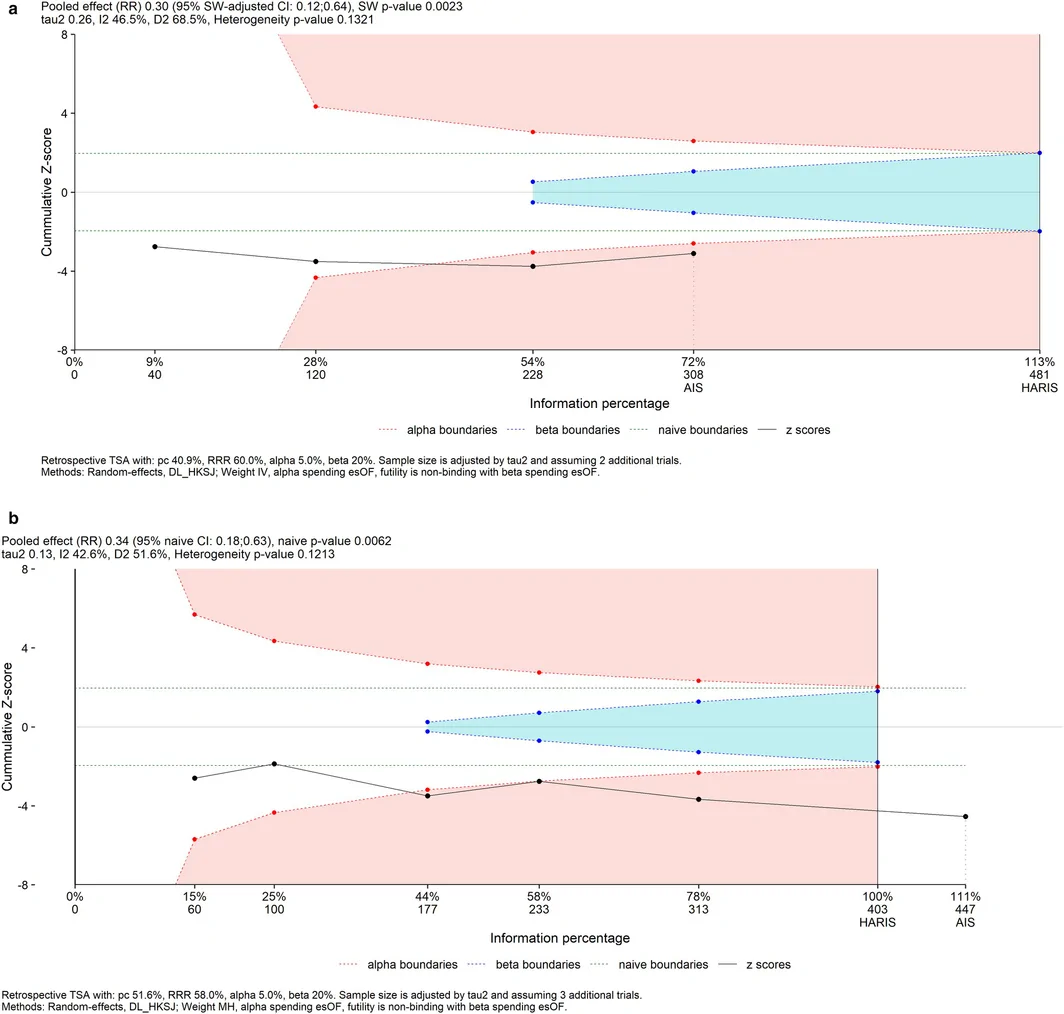
Trial Sequential Analysis (TSA). (a) Trial Sequential Analysis for Emergence Delirium at the 0.25 mg/kg Esketamine Dose. (b) Trial Sequential Analysis for Emergence Delirium at the 0.5 mg/kg Esketamine Dose.

### 3.5 Sensitivity Analysis

The leave‐one‐out sensitivity analysis demonstrated stable effect sizes for emergence delirium, pain scores, and PACU stay (Figures S23–S25). In contrast, the effect size for extubation time was influenced by the exclusion of the Zhaoxia 2022 arms and the Wu 2025–0.5 mg/kg arm; moreover, the effect size for PONV was altered upon omission of either Xu 2025 arms, the Jiang 2025–0.1 mg/kg arm, or the Zhaoxia 2022–0.75 mg/kg arm (Figures S26 and S27).

### 3.6 Publication Bias and Certainty of Evidence

Emergence delirium demonstrated potential publication bias, with Egger's test *p* value < 0.001 (Figure S28). Trim‐and‐fill imputed 4 missing studies, shifting the pooled RR from 0.35 (95% CI, 0.23–0.53) to 0.45 (95% CI, 0.28–0.72), while the association remained significant in favor of esketamine (Figure S33). No evidence of publication bias was observed across all assessed outcomes, including extubation time (Figures S29–S32). The certainty of evidence was rated as moderate for ED and all other outcomes, except for extubation time, which was rated as low. Table S6 presents the summary of findings and detailed certainty ratings.

## 4 Discussion

The present meta‐analysis aims to evaluate the effect of esketamine on ED following sevoflurane‐based general anesthesia in pediatric surgical procedures. Across the pooled evidence, esketamine consistently reduced the risk of ED with moderate certainty, regardless of dose category (subanesthetic or anesthetic), specific dosing regimens (0.25 or 0.5), route and timing of administration (intranasal or intravenous), or type of surgery (ophthalmic or otolaryngologic). Esketamine was associated with moderate to large reductions in postoperative pain scores across anesthetic levels and dose subgroups. However, in the route‐of‐administration subgroup analysis, this effect was statistically significant with intravenous administration and was also observed with the intranasal route. Moreover, the overall pooled analysis showed a slight prolongation of extubation time, particularly in patients receiving an anesthetic dose, whereas no significant effect was seen at subanesthetic doses. Across all settings, esketamine did not affect PACU length of stay. Additionally, the incidence of PONV was significantly reduced overall with esketamine. However, this significance was not maintained across subgroups, except for the intravenous route, likely due to limited statistical power.

The comprehensive mechanism underlying ED remains incompletely understood, although several perioperative risk factors have been implicated in its pathophysiology [46]. Sevoflurane is considered a contributing factor, owing to its low blood gas partition coefficient, which allows rapid induction but equally rapid emergence [7, 47, 48, 49]. This abrupt transition may disrupt the reestablishment of balance between inhibitory and excitatory neural systems [50]. It has also been proposed that sevoflurane produces an asynchrony in the recovery of different brain regions, with sensorimotor pathways regaining function before higher cognitive networks [51]. Such heterogeneous clearance of inhalational agents may produce a transient state of neural hyperexcitability, manifesting clinically as ED [52]. Esketamine is well‐positioned to counteract this excitatory state through potent NMDA receptor antagonism, which prevents glutamate binding to NMDA receptors and thereby attenuates the excitatory surge [53]. A more pronounced inhibition of this pathway is anticipated to yield superior control over neuronal excitability. However, our subgroup analysis revealed no significant differences in anesthetic levels across esketamine doses, and meta‐regression did not establish a significant association between the magnitude of risk reduction and esketamine dose. These observations suggest that subanesthetic doses may sufficiently block NMDA receptors to modulate hyperexcitable neural circuits, without additional therapeutic benefit from escalating to anesthetic doses. This dose‐independent effect highlights esketamine's versatile clinical profile. Our findings suggest that subanesthetic doses (≤ 0.3 mg/kg) may provide sufficient NMDA receptor antagonism to attenuate the neuronal hyperexcitability associated with sevoflurane emergence. The preventive effect on ED therefore appears achievable through neuromodulatory and analgesic mechanisms even at lower doses, without requiring escalation to anesthetic‐dose regimens (≥ 0.5 mg/kg). This observation is further supported by the absence of significant differences between dose subgroups in both subgroup analysis and meta‐regression.

Similarly, the absence of significant differences across surgical specialties may indicate that esketamine provides consistent protection across pediatric surgical settings with elevated ED risk.

Pain is also recognized as a contributing factor to ED, largely due to its capacity to trigger a sympathetic reaction in response to physiological distress [54, 55]. This effect may be particularly pronounced following discontinuation of sevoflurane, as its rapid washout eliminates any residual analgesic effect, resulting in heightened sympathetic tone and increased perioperative distress. Esketamine provides analgesia by modulating nociceptive transmission at the dorsal horn, thereby attenuating sympathetic activation and reducing anxiety, which may account for the significant reductions in pain scores observed in our analysis [56, 57]. Our findings of simultaneous reductions in both pain scores and ED suggest that esketamine's anti‐agitation effect may be partially mediated by its potent analgesic properties, addressing the clinical overlap where postoperative pain is often miscategorized as delirium. Furthermore, this beneficial analgesic effect was consistently observed in the subgroups receiving intranasal administration. The relatively low bioavailability of intranasal esketamine, which is approximately 48% or less, may provide a plausible explanation, influenced by variability in drug formulation, delivery devices, and insufflation techniques [58]. Such factors may limit systemic exposure to therapeutic levels sufficient to produce analgesic and anti‐agitation effects.

Further patient‐related factors have been proposed to influence the occurrence of ED, including age and body weight [49, 59, 60]. However, our meta‐regression did not find age or weight to be significant modifiers of the treatment effect. The absence of a relationship between weight and ED risk reduction may suggest a lack of strong biological influence of this parameter on esketamine pharmacodynamics across the included pediatric populations.

However, the duration of anesthesia demonstrated an inverse relationship with ED risk ratio, indicating that longer procedures were associated with greater preventive effects, whereas no association was observed with surgical duration. This observation may reflect the role of rapid emergence as a principal precipitating factor of agitation [61]. Nonetheless, this finding should be interpreted with caution, as the data were not well distributed and existing literature has reported that patients experiencing ED were often exposed to significantly prolonged anesthesia and surgical durations [62].

In contrast to its racemate, esketamine showed no increase in PONV and demonstrated a statistically significant reduction in the overall pooled analysis [63]. However, this effect was not consistently observed across dose subgroups, likely due to insufficient statistical power, whereas a significant reduction was observed with intravenous administration. This may be attributable to the analgesic properties of esketamine, which reduce perioperative opioid requirements as a recognized contributor to PONV, as supported by Wang et al. [64].

Recent meta‐analyses by Li et al. and Liu et al. also evaluated the effect of esketamine on ED among children. They reported a significant risk reduction exceeding 60%, which aligns with our findings [44]. However, neither analysis provided conclusions specific to sevoflurane. Notably, Li et al. further demonstrated that esketamine exerted a protective effect regardless of the route of administration or the timing of a single bolus dose, findings that align with our results [44]. It is worth noting that studies employing continuous esketamine infusion were excluded from our analysis to reduce heterogeneity across dosing regimens. However, Li et al. reported that continuous infusion throughout anesthesia did not reach statistical significance, although a clear trend toward benefit was observed, likely owing to limited precision and the small sample size from only two included studies [44].

In addition, both studies support our findings by reporting no statistically significant differences in PACU length of stay. In the study by Liu et al., esketamine consistently reduced ED at both sub‐anesthetic and anesthetic doses, demonstrating superiority over placebo, opioids, and other anesthetic agents [65]. Although an anesthetic‐level subgroup analysis was performed, further dose‐specific investigation is warranted, particularly given the influence of dosing heterogeneity demonstrated in our study [65]. They also found that esketamine was associated with a significant reduction in postoperative pain scores, indicating meaningful analgesic efficacy [65]. Additionally, the safety profile of esketamine was assessed broadly, without detailing the types of adverse events, which limited the interpretability of the conclusions despite statistical significance [65].

A recent meta‐analysis by Yang et al. [24] also reported esketamine's benefits; however, our study addresses several of its limitations. While their meta‐analysis included heterogeneous control groups and various anesthetics, our focus on placebo‐controlled trials and sevoflurane‐based Anesthesia provides clearer, more targeted clinical insights. Crucially, we incorporate a substantial portion of newly published RCTs from 2025 and employ TSA to assess the strength of the cumulative evidence. While the TSA suggests the required information size has been reached, this result should be interpreted carefully. The presence of potential publication bias and heterogeneity suggests that these findings, while providing a stronger evidence base than previous reviews, require further validation in larger, more diverse cohorts.

Opioids have also demonstrated promising control of ED in children; however, this benefit comes at the cost of prolonged PACU stay and increased PONV [11]. Moreover, the addition of dexmedetomidine appears to potentiate the risk‐reduction effect of esketamine [13]. These findings suggest that esketamine offers a superior efficacy and safety profile compared with opioids and indicate that combination strategies may provide enhanced effectiveness in sevoflurane‐based anesthesia settings [66].

Propofol‐based Anesthesia is widely recognized as an effective strategy for reducing ED incidence in pediatric patients, although sevoflurane remains commonly used because of its rapid induction and recovery characteristics [67]. In this context, our findings suggest that esketamine may represent a valuable adjunctive strategy for mitigating ED risk specifically in sevoflurane‐based anesthesia settings.

This meta‐analysis has several notable strengths, including the use of rigorous methodological approaches to explore robustness, heterogeneity, and dose–response relationships, supported by subgroup analyses, meta‐regression, leave‐one‐out sensitivity analyses, and trial sequential analysis. These complementary methods consistently demonstrated a stable and clinically meaningful reduction in ED risk exceeding 60%, reinforcing confidence in the reliability of the findings despite between‐study variability. However, several limitations warrant consideration. Although efforts were made to evaluate esketamine in standardized anesthetic settings, some studies included premedication or additional anesthetic agents alongside sevoflurane. Variations in ED assessment tools and cutoff thresholds also introduced potential measurement heterogeneity.

Nevertheless, the ED sensitivity analysis demonstrated remarkable stability, with risk reductions consistently exceeding 60% across all leave‐one‐out iterations. Double‐counting of shared control groups was unavoidable because some studies included multiple esketamine arms. However, this may have led to a slight artificial narrowing of the confidence intervals. Trial sequential analysis at both 0.25 and 0.5 mg/kg doses, unaffected by the double‐counting issue, confirmed the robustness of the effect. A significant limitation of this meta‐analysis is that all included randomized controlled trials were conducted exclusively within Chinese pediatric centers. This geographical homogeneity raises substantial concerns regarding the external validity and generalizability of our findings. Variations in genetic factors, differences in perioperative clinical protocols, and diverse cultural approaches to pediatric behavioral assessment in other global regions may influence the observed efficacy and safety of esketamine. Consequently, our results must be interpreted with caution when applied to non‐Chinese populations, and international multicenter trials are essential to confirm the generalizability of these conclusions worldwide.

In summary, this meta‐analysis supports the use of esketamine to prevent ED and improve postoperative pain management in children undergoing sevoflurane‐based anesthesia, demonstrating a favorable efficacy and safety profile. While the underlying mechanisms of ED remain incompletely understood, further research is needed to elucidate the pathophysiology and optimize targeted interventions.

## Author Contributions

All authors read and approved the final manuscript. **Munder Lateiresh:** conceptualization, methodology, investigation, data curation, formal analysis, writing‐original draft, writing‐review, and editing. **Abdelaziz Louat:** conceptualization, methodology, investigation, data curation, writing‐original draft, writing‐review, and editing. **Mukhtar Alwefati:** investigation, data curation, writing‐original draft, writing‐review, and editing. **Alhasan Altayf:** investigation, data curation, formal analysis. **Matthieu Legrand:** investigation, writing‐original draft, writing‐review, and editing. **Phillip D. Cohen:** investigation, writing‐original draft, writing‐review, and editing. **Eman Abdulwahed:** investigation, data curation, writing, review, and editing. **Sapna R. Kudchadkar:** validation, writing, review, and editing. **Muhammed Elhadi:** conceptualization, investigation, supervision, analysis, project administration, writing‐review, and editing.

## Funding

This study was supported by Korea University.

## Ethics Statement

The authors have nothing to report.

## Conflicts of Interest

The authors declare no conflicts of interest.

## Supporting information


**Table S1:** Abstract PRISMA 2020 checklist.
**Table S2:** Main‐text PRISMA 2020 checklist.
**Table S3:** AMSTAR 2 checklist.
**Table S4:** Search strategy table.
**Table S5:** Outcome definitions table.
**Table S6:** Summary of findings table with GRADE assessment.
**Figure S1:** Traffic light plot.
**Figure S2:** Summary plot.
**Figure S3:** Overall emergence of delirium.
**Figure S4:** Subgroup analysis of the emergence of delirium based on route of administration.
**Figure S5:** Subgroup analysis of the emergence of delirium based on the time of administration.
**Figure S6:** Subgroup analysis of the emergence of delirium based on the type of surgery.
**Figure S7:** Subgroup analysis of pain scores based on subanesthetic versus anesthetic dose.
**Figure S8:** Overall pain scores.
**Figure S9:** Subgroup analysis of pain scores based on specific doses.
**Figure S10:** Subgroup analysis of pain scores based on route of administration.
**Figure S11:** Subgroup analysis of extubation time based on subanesthetic versus anesthetic dose.
**Figure S12:** Overall extubation time.
**Figure S13:** Subgroup analysis of extubation time based on specific doses.
**Figure S14:** Subgroup analysis of extubation time based on route of administration.
**Figure S15:** Subgroup analysis of PACU stay based on subanesthetic versus anesthetic dose.
**Figure S16:** Overall PACU stay.
**Figure S17:** Subgroup analysis of PACU stay based on specific doses.
**Figure S18:** Subgroup analysis of PACU stay based on route of administration.
**Figure S19:** Subanesthetic versus anesthetic subgroup analysis for postoperative nausea and vomiting.
**Figure S20:** Overall, postoperative nausea and vomiting.
**Figure S21:** Subgroup analysis of postoperative nausea and vomiting based on specific doses.
**Figure S22:** Subgroup analysis of postoperative nausea and vomiting based on route of administration.
**Figure S23:** Leave‐one‐out sensitivity analysis for emergence delirium.
**Figure S24:** Leave‐one‐out sensitivity analysis for pain scores.
**Figure S25:** Leave‐one‐out sensitivity analysis for PACU stay.
**Figure S26:** Leave‐one‐out sensitivity analysis for extubation time.
**Figure S27:** Leave‐one‐out sensitivity analysis for postoperative nausea and vomiting.
**Figure S28:** Contour‐enhanced funnel plot for emergence delirium.
**Figure S29:** Contour‐enhanced funnel plot for pain scores.
**Figure S30:** Contour‐enhanced funnel plot for extubation time.
**Figure S31:** Contour‐enhanced funnel plot for PACU stay.
**Figure S32:** Contour‐enhanced funnel plot for postoperative nausea and vomiting.
**Figure S33:** Trim and fill funnel plot of emergence delirium.

## Data Availability

All data included in this meta‐analysis were extracted from previously published studies. Any requests for underlying datasets should be directed to the corresponding authors of the original publications.
