# Supplementary material for: Effects of Esketamine on Emergence Delirium in Children Undergoing Sevoflurane Anesthesia: A Systematic Review, Meta‐Analysis, and Trial Sequential Analysis
Source: Paediatr Anaesth. 2026 Aug 6;36(11):1361–74. doi: 10.1002/pan.70272 (PMC13639193; doi:10.1002/pan.70272)
Supplement: Supplementary file 1 [file PAN-36-1361-s001.docx]

**Supplementary Appendix**

*This supplementary appendix for the article entitled:*

**“Effects of Esketamine on Emergence Delirium in Children Undergoing Sevoflurane Anesthesia:**

**A Systematic Review, Meta-Analysis, and Trial Sequential Analysis”**

**Table of Contents:**

[Supplementary Tables	3](#_Toc229250937)

[**Table S1.** *Abstract PRISMA 2020 checklist*	3](#_Toc229250938)

[**Table S2.** *Main-text PRISMA 2020 checklist*	4](#_Toc229250939)

[**Table S3.** *AMSTAR 2 checklist*	7](#_Toc229250940)

[**Table S4.** *Search strategy table*	11](#_Toc229250941)

[**Table S5.** *Outcomes definitions table*	12](#_Toc229250942)

[**Table S6:** *Summary findings table with GRADE assessment*	14](#_Toc229250943)

[Supplementary Figures	16](#_Toc229250944)

[Quality Assessment	16](#_Toc229250945)

[**Figure S1.** *Traffic light plot*	16](#_Toc229250946)

[**Figure S2.** *Summary plot*	16](#_Toc229250947)

[Forest Plots	17](#_Toc229250948)

[**Figure S3.** *Overall emergence of delirium*	17](#_Toc229250949)

[**Figure S4.** *Subgroup analysis of the emergence of delirium based on route of administration*	18](#_Toc229250950)

[**Figure S5.** *Subgroup analysis of the emergence of delirium based on time of administration*	19](#_Toc229250951)

[**Figure S6.** *Subgroup analysis of the emergence of delirium based on the type of surgery*	20](#_Toc229250952)

[**Figure S7.** *Subgroup analysis of pain scores based on subanesthetic versus anesthetic dose*	21](#_Toc229250953)

[**Figure S8.** *Overall pain scores*	22](#_Toc229250954)

[**Figure S9.** *Subgroup analysis of pain scores based on specific doses*	23](#_Toc229250955)

[**Figure S10.** *Subgroup analysis of pain scores based on route of administration*	24](#_Toc229250956)

[**Figure S11.** *Subgroup analysis of extubation time based on subanesthetic versus anesthetic dose*	25](#_Toc229250957)

[**Figure S12.** *Overall extubation time*	26](#_Toc229250958)

[**Figure S13.** *Subgroup analysis of extubation time based on specific doses*	26](#_Toc229250959)

[**Figure S14.** *Subgroup analysis of extubation time based on route of administration*	27](#_Toc229250960)

[**Figure S15.** *Subgroup analysis of PACU based on subanesthetic versus anesthetic dose*	28](#_Toc229250961)

[**Figure S16.** *Overall PACU stay*	29](#_Toc229250962)

[**Figure S17.** *Subgroup analysis of PACU based on specific doses*	30](#_Toc229250963)

[**Figure S18.** *Subgroup analysis of PACU based on route of administration*	31](#_Toc229250964)

[**Figure S19.** *Subanesthetic versus anesthetic subgroup analysis for PONV*	32](#_Toc229250965)

[**Figure S20.** *Overall PONV*	33](#_Toc229250966)

[**Figure S21.** *Subgroup analysis of PONV based on specific doses*	34](#_Toc229250967)

[**Figure S22.** *Subgroup analysis of PONV based on route of administration*	35](#_Toc229250968)

[Sensitivity Analysis	36](#_Toc229250969)

[**Figure S23.** *Overall leave-one-out sensitivity analysis for the emergence delirium*	36](#_Toc229250970)

[**Figure S24.** *Overall leave-one-out sensitivity analysis for pain scores*	36](#_Toc229250971)

[**Figure S25.** *Overall leave-one-out sensitivity analysis for PACU*	37](#_Toc229250972)

[**Figure S26.** *Overall leave-one-out sensitivity analysis for extubation time*	37](#_Toc229250973)

[**Figure S27.** *Overall leave-one-out sensitivity analysis for PONV*	38](#_Toc229250974)

[Funnel Plots	39](#_Toc229250975)

[**Figure S28.** *Overall contour-enhanced funnel plot for the emergence delirium*	39](#_Toc229250976)

[**Figure S29.** *Overall contour-enhanced funnel plot for pain scores*	40](#_Toc229250977)

[**Figure S30.** *Overall contour-enhanced funnel plot for extubation time*	41](#_Toc229250978)

[**Figure S31.** *Overall contour-enhanced funnel plot for PACU stay*	42](#_Toc229250979)

[**Figure S32.** *Overall contour-enhanced funnel plot for PONV*	43](#_Toc229250980)

[**Figure S33.** *Trim and fill funnel plot of emergence delirium.*	44](#_Toc229250981)

# **Supplementary Tables**

### **Table S1.** *Abstract PRISMA 2020 checklist*

| **Section and Topic** | **Item #** | **Checklist item** | **Reported (Yes/No)** |
| --- | --- | --- | --- |
| **TITLE** | | |  |
| Title | 1 | Identify the report as a systematic review. | Yes |
| **BACKGROUND** | | |  |
| Objectives | 2 | Provide an explicit statement of the main objective(s) or question(s) the review addresses. | Yes |
| **METHODS** | | |  |
| Eligibility criteria | 3 | Specify the inclusion and exclusion criteria for the review. | Yes |
| Information sources | 4 | Specify the information sources (e.g. databases, registers) used to identify studies and the date when each was last searched. | Yes |
| Risk of bias | 5 | Specify the methods used to assess risk of bias in the included studies. | Yes |
| Synthesis of results | 6 | Specify the methods used to present and synthesise results. | Yes |
| **RESULTS** | | |  |
| Included studies | 7 | Give the total number of included studies and participants and summarise relevant characteristics of studies. | Yes |
| Synthesis of results | 8 | Present results for main outcomes, preferably indicating the number of included studies and participants for each. If meta-analysis was done, report the summary estimate and confidence/credible interval. If comparing groups, indicate the direction of the effect (i.e. which group is favoured). | Yes |
| **DISCUSSION** | | |  |
| Limitations of evidence | 9 | Provide a brief summary of the limitations of the evidence included in the review (e.g. study risk of bias, inconsistency and imprecision). | Yes |
| Interpretation | 10 | Provide a general interpretation of the results and important implications. | yes |
| **OTHER** | | |  |
| Funding | 11 | Specify the primary source of funding for the review. | NA |
| Registration | 12 | Provide the register name and registration number. | Yes |

### **Table S2.** *Main-text PRISMA 2020 checklist*

| **Section/Topic** | **Item #** | **Prisma 2020 Checklist item for manuscript** | **Location** |
| --- | --- | --- | --- |
| **TITLE** | | |  |
| Title | 1 | Identify the report as a systematic review. | Page 1 |
| **ABSTRACT** | | |  |
| Abstract | 2 | See the PRISMA 2020 for Abstracts checklist. | Page 3 |
| **INTRODUCTION** | | |  |
| Rationale | 3 | Describe the rationale for the review in the context of existing knowledge. | Page 4 |
| Objectives | 4 | Provide an explicit statement of the objective(s) or question(s) the review addresses. | Page 4 |
| **METHODS** | | |  |
| Eligibility criteria | 5 | Specify the inclusion and exclusion criteria for the review and how studies were grouped for the syntheses. | Page 5-6 |
| Information sources | 6 | Specify all databases, registers, websites, organisations, reference lists and other sources searched or consulted to identify studies. Specify the date when each source was last searched or consulted. | Page 6 |
| Search strategy | 7 | Present the full search strategies for all databases, registers and websites, including any filters and limits used. | Table S4 |
| Selection process | 8 | Specify the methods used to decide whether a study met the inclusion criteria of the review, including how many reviewers screened each record and each report retrieved, whether they worked independently, and if applicable, details of automation tools used in the process. | Page 5 |
| Data collection process | 9 | Specify the methods used to collect data from reports, including how many reviewers collected data from each report, whether they worked independently, any processes for obtaining or confirming data from study investigators, and if applicable, details of automation tools used in the process. | Page 6 |
| Data items | 10a | List and define all outcomes for which data were sought. Specify whether all results that were compatible with each outcome domain in each study were sought (e.g. for all measures, time points, analyses), and if not, the methods used to decide which results to collect. | Table S5 |
|  | 10b | List and define all other variables for which data were sought (e.g. participant and intervention characteristics, funding sources). Describe any assumptions made about any missing or unclear information. | Page 6 |
| Study risk of bias assessment | 11 | Specify the methods used to assess risk of bias in the included studies, including details of the tool(s) used, how many reviewers assessed each study and whether they worked independently, and if applicable, details of automation tools used in the process. | Page 6 |
| Effect measures | 12 | Specify for each outcome the effect measure(s) (e.g. risk ratio, mean difference) used in the synthesis or presentation of results. | Page 7 |
| Synthesis methods | 13a | Describe the processes used to decide which studies were eligible for each synthesis (e.g. tabulating the study intervention characteristics and comparing against the planned groups for each synthesis (item #5)). | Page 7-8 |
|  | 13b | Describe any methods required to prepare the data for presentation or synthesis, such as handling of missing summary statistics, or data conversions. | Page 7 |
|  | 13c | Describe any methods used to tabulate or visually display results of individual studies and syntheses. | Page 8-9 |
|  | 13d | Describe any methods used to synthesize results and provide a rationale for the choice(s). If meta-analysis was performed, describe the model(s), method(s) to identify the presence and extent of statistical heterogeneity, and software package(s) used. | Page 7-8 |
|  | 13e | Describe any methods used to explore possible causes of heterogeneity among study results (e.g. subgroup analysis, meta-regression). | Page 8 |
|  | 13f | Describe any sensitivity analyses conducted to assess robustness of the synthesized results. | Page 8 |
| Reporting bias assessment | 14 | Describe any methods used to assess risk of bias due to missing results in a synthesis (arising from reporting biases). | Page 8 |
| Assessment | 15 | Describe any Certainty methods used to assess certainty (or confidence) in the body of evidence for an outcome. | Page 7; Table S5 |
| **RESULTS** | | |  |
| Study selection | 16a | Describe the results of the search and selection process, from the number of records identified in the search to the number of studies included in the review, ideally using a flow diagram. | Page 9; Figure 1 |
|  | 16b | Cite studies that might appear to meet the inclusion criteria, but which were excluded, and explain why they were excluded. | Page 9 |
| Study characteristics | 17 | Cite each included study and present its characteristics. | Page 9; Table 1 |
| Risk of bias in studies | 18 | Present assessments of risk of bias for each included study. | Page 9; Figures S1-S2 |
| Results of individual studies | 19 | For all outcomes, present, for each study: (a) summary statistics for each group (where appropriate) and (b) an effect estimate and its precision (e.g. confidence/credible interval), ideally using structured tables or plots. | Page 9 Supplementary figures S3 to S22 |
| Results of syntheses | 20a | For each synthesis, briefly summarise the characteristics and risk of bias among contributing studies. | Page 9-10 |
|  | 20b | Present results of all statistical syntheses conducted. If meta-analysis was done, present for each the summary estimate and its precision (e.g. confidence/credible interval) and measures of statistical heterogeneity. If comparing groups, describe the direction of the effect. | Page 10-11 |
|  | 20c | Present results of all investigations of possible causes of heterogeneity among study results. |  |
|  | 20d | Present results of all sensitivity analyses conducted to assess the robustness of the synthesized results. | Supplementary Figures S23 to S27 |
| Reporting biases | 21 | Present assessments of risk of bias due to missing results (arising from reporting biases) for each synthesis assessed. | Supplementary Figures S28 and S32 |
| Certainty of evidence | 22 | Present assessments of certainty (or confidence) in the body of evidence for each outcome assessed. | Table S6 |
| **DISCUSSION** | | |  |
| Discussion | 23a | Provide a general interpretation of the results in the context of other evidence. | Page 13-16 |
|  | 23b | Discuss any limitations of the evidence included in the review. | Page 17 |
|  | 23c | Discuss any limitations of the review processes used. | Page 17 |
|  | 23d | Discuss implications of the results for practice, policy, and future research. | Page 17 |
| **OTHER INFORMATION** | | |  |
| Registration and protocol | 24a | Provide registration information for the review, including register name and registration number, or state that the review was not registered. | Page 5 |
|  | 24b | Indicate where the review protocol can be accessed, or state that a protocol was not prepared. | Page 5 |
|  | 24c | Describe and explain any amendments to information provided at registration or in the protocol. |  |
| Support | 25 | Describe sources of financial or non-financial support for the review, and the role of the funders or sponsors in the review. | Page 21 |
| Competing interests | 26 | Declare any competing interests of review authors. | Page 21 |
| Availability of data, code and other materials | 27 | Report which of the following are publicly available and where they can be found: template data collection forms; data extracted from included studies; data used for all analyses; analytic code; any other materials used in the review. | Page 21 |

### **Table S3.** *AMSTAR 2 checklist*

| **1. Did the research questions and inclusion criteria for the review include the components of PICO?** | | | | |
| --- | --- | --- | --- | --- |
| For Yes:  ✓ Population  ✓ Intervention  ✓ Comparator group  ✓ Outcome | | Optional (recommended)   Timeframe for follow-up | ✓ Yes   No |  |
| **2. Did the report of the review contain an explicit statement that the review methods were established prior to the conduct of the review and did the report justify any significant deviations from the protocol?** | | | | |
|  | For Partial Yes:  The authors state that they had a written protocol or guide that included ALL the following:   review question(s)   a search strategy   inclusion/exclusion criteria   a risk of bias assessment | For Yes:  As for partial yes, plus the protocol should be registered and should also have specified:  ✓ a meta-analysis/synthesis plan, if appropriate, *and*  ✓ a plan for investigating causes of heterogeneity  ✓ justification for any deviations from the protocol | ✓ Yes   Partial Yes   No |  |
| **3. Did the review authors explain their selection of the study designs for inclusion in the review?** | | | | |
|  | For Yes, the review should satisfy ONE of the following:  ✓ *Explanation for* including only RCTs   OR *Explanation for* including only NRSI   OR *Explanation for* including both RCTs and NRSI | | ✓ Yes   No |  |
| **4. Did the review authors use a comprehensive literature search strategy?** | | | | |
|  | For Partial Yes (all the following):   searched at least 2 databases (relevant to research question)   provided key word and/or search strategy   justified publication restrictions (e.g. language) | For Yes, should also have (all the following):  ✓ searched the reference lists / bibliographies of included studies  ✓ searched trial/study registries  ✓ included/consulted content experts in the field  ✓ where relevant, searched for grey literature  ✓ conducted search within 24 months of completion of the review | ✓ Yes   Partial Yes   No |  |
|  | **5. Did the review authors perform study selection in duplicate?** | | |  |
|  | For Yes, either ONE of the following:  ✓ at least two reviewers independently agreed on selection of eligible studies and achieved consensus on which studies to include   OR two reviewers selected a sample of eligible studies and achieved good agreement (at least 80 percent), with the remainder selected by one reviewer. | | ✓ Yes   No |  |

| **6. Did the review authors perform data extraction in duplicate?** | | | |
| --- | --- | --- | --- |
| For Yes, either ONE of the following:  ✓ at least two reviewers achieved consensus on which data to extract from included studies  • OR two reviewers extracted data from a sample of eligible studies and achieved good agreement (at least 80 percent), with the remainder extracted by one reviewer. | | | ✓ Yes  • No |
| **7. Did the review authors provide a list of excluded studies and justify the exclusions?** | | | |
|  | For Partial Yes:  • provided a list of all potentially relevant studies that were read  in full-text form but excluded from the review | For Yes, must also have:  ✓ Justified the exclusion from the review of each potentially relevant study | ✓ Yes  • Partial Yes  • No |
| **8. Did the review authors describe the included studies in adequate detail?** | | | |
|  | For Partial Yes (ALL the following):  ✓ described populations  ✓ described interventions  ✓ described comparators  ✓ described outcomes  ✓ described research designs | For Yes, should also have ALL the following:  • described population in detail  • described intervention in detail (including doses where relevant)  • described comparator in detail (including doses where relevant)  • described study’s setting  • timeframe for follow-up | • Yes  ✓ Partial Yes  • No |
| **9. Did the review authors use a satisfactory technique for assessing the risk of bias (RoB) in individual studies that were included in the review?** | | | |
|  | **RCTs**  For Partial Yes, must have assessed RoB from  • unconcealed allocation, *and*  • lack of blinding of patients and assessors when assessing outcomes (unnecessary for objective outcomes such as all-  cause mortality) | For Yes, must also have assessed RoB from:  ✓ allocation sequence that was not truly random, *and*  ✓ selection of the reported result from among multiple measurements or analyses of a specified outcome | ✓ Yes  • Partial Yes  • No  • Includes only NRSI |
|  | **NRSI**  For Partial Yes, must have assessed RoB:  • from confounding, *and*  • from selection bias | For Yes, must also have assessed RoB:  • methods used to ascertain exposures and outcomes, *and*  • selection of the reported result from among multiple measurements or analyses of a specified outcome | • Yes  • Partial Yes  • No  • Includes only RCTs |
|  | **10. Did the review authors report on the sources of funding for the studies included in the review?** | | |
|  | For Yes  ✓ Must have reported on the sources of funding for individual studies included ✓ Yes in the review. Note: Reporting that the reviewers looked for this information • No but it was not reported by study authors also qualifies | | |

| **11. If meta-analysis was performed did the review authors use appropriate methods for statistical combination of results?** | | | |
| --- | --- | --- | --- |
|  | **RCTs**  For Yes:  ✓ The authors justified combining the data in a meta-analysis  ✓ AND they used an appropriate weighted technique to combine study results and adjusted for heterogeneity if present.  ✓ AND investigated the causes of any heterogeneity | ✓ Yes  • No  • No meta-analysis conducted |  |
|  | **For NRSI**  For Yes:  • The authors justified combining the data in a meta-analysis  • AND they used an appropriate weighted technique to combine study results, adjusting for heterogeneity if present  • AND they statistically combined effect estimates from NRSI that were adjusted for confounding, rather than combining raw data, or justified combining raw data when adjusted effect estimates were not available  • AND they reported separate summary estimates for RCTs and NRSI separately when both were included in the review | • Yes  • No  • No meta-analysis conducted |  |
| **12. If meta-analysis was performed, did the review authors assess the potential impact of RoB in individual studies on the results of the meta-analysis or other evidence synthesis?** | | | |
|  | For Yes:  • included only low risk of bias RCTs  ✓ OR, if the pooled estimate was based on RCTs and/or NRSI at variable RoB, the authors performed analyses to investigate possible impact of RoB on summary estimates of effect. | ✓ Yes  • No  • No meta-analysis conducted |  |
| **13. Did the review authors account for RoB in individual studies when interpreting/ discussing the results of the review?** | | | |
|  | For Yes:  • included only low risk of bias RCTs  ✓ OR, if RCTs with moderate or high RoB, or NRSI were included the review provided a discussion of the likely impact of RoB on the results | ✓ Yes  • No |  |
| **14. Did the review authors provide a satisfactory explanation for, and discussion of, any heterogeneity observed in the results of the review?** | | | |
|  | For Yes:  • There was no significant heterogeneity in the results  ✓ OR if heterogeneity was present the authors performed an investigation of sources of any heterogeneity in the results and discussed the impact of this on the results of the review | ✓ Yes  • No |  |
| **15. If they performed quantitative synthesis did the review authors carry out an adequate investigation of publication bias (small study bias) and discuss its likely impact on the results of the review?** | | | |
|  | For Yes:  • performed graphical or statistical tests for publication bias and discussed the likelihood and magnitude of impact of publication bias | • Yes  ✓ No  • No meta-analysis conducted |  |

| **16. Did the review authors report any potential sources of conflict of interest, including any funding they received for conducting the review?** | | | |
| --- | --- | --- | --- |
|  | For Yes:  ✓ The authors reported no competing interests OR  • The authors described their funding sources and how they managed potential conflicts of interest | ✓ Yes  • No |  |

### **Table S4.** *Search strategy table*

| Database | Search Strategy | Results |
| --- | --- | --- |
| PubMed  Date:  *27 March 2026* | (("Infant, Newborn"[Mesh]) OR ("Infant"[Mesh]) OR ("Child"[Mesh]) OR ("Child, Preschool"[Mesh]) OR ("Adolescent"[Mesh]) OR (infant*) OR (neonate*) OR (newborn*) OR (child*) OR (preschool*) OR (adolescent*) OR (pediatric*) OR (paediatric*))  AND  (("Ketamine"[Mesh]) OR (esketamin*) OR ((S)-ketamine) OR ("Esketamine") OR (Spravato) OR (Ketanest))  AND  (("Sevoflurane"[Mesh]) OR (sevoflurane) OR (Sevorane)) | **201** |
| Scopus  Date:  *27 March 2026* | ( ( infant OR infants OR neonate OR neonates OR newborn OR newborns OR child OR children OR preschool OR preschoolers OR adolescent OR adolescents OR pediatric OR pediatrics OR paediatric OR paediatrics ) AND ( esketamine OR "Esketamine" OR "S ketamine" OR Spravato OR Ketanest ) AND ( sevoflurane OR Sevorane ) ) | **1198** |
| WOS  *(No filter applied)*  Date:  *27 March 2026* | ((infant) OR (infants) OR (neonate) OR (neonates) OR (newborn) OR (newborns) OR (child) OR (children) OR (preschool) OR (preschoolers) OR (adolescent) OR (adolescents) OR (pediatric) OR (pediatrics) OR (paediatric) OR (paediatrics)) AND ((esketamine) OR ("Esketamine") OR ((S)-ketamine) OR (spianato) OR (ketanes)) AND ((sevoflurane) OR (severance)) | **209** |
| Embase  *(Search by title, abstract, and keyword)*  Date:  *27 March 2026* | (infant:ti,ab,kw OR infants:ti,ab,kw OR neonate:ti,ab,kw OR neonates:ti,ab,kw OR newborn:ti,ab,kw OR newborns:ti,ab,kw OR child:ti,ab,kw OR children:ti,ab,kw OR preschool:ti,ab,kw OR preschoolers:ti,ab,kw OR adolescent:ti,ab,kw OR adolescents:ti,ab,kw OR pediatric:ti,ab,kw OR pediatrics:ti,ab,kw OR paediatric:ti,ab,kw OR paediatrics:ti,ab,kw)  AND  (esketamine:ti,ab,kw OR 'Esketamine':ti,ab,kw OR (s:ti,ab,kw AND -ketamine:ti,ab,kw) OR spravato:ti,ab,kw OR ketanest:ti,ab,kw)  AND  (sevoflurane:ti,ab,kw OR sevorane:ti,ab,kw) | **139** |
| Cochrane library  Date:  *27 March 2026* | ((infant) OR (infants) OR (neonate) OR (neonates) OR (newborn) OR (newborns) OR (child) OR (children) OR (preschool) OR (preschoolers) OR (adolescent) OR (adolescents) OR (pediatric) OR (pediatrics) OR (paediatric) OR (paediatrics))  AND  ((esketamine) OR ("Esketamine") OR ((S)-ketamine) OR (Spravato) OR (Ketanest))  AND  ((sevoflurane) OR (Sevorane)) | **30** |
| Google Scholar  *(Via Publish or Perish)*  Date:  *27 March 2026* | **allintitle 1:** (Esketamine) AND (Anesthesia) | **172** |
|  | **allintitle 2:** (Esketamine) AND (Sevoflurane) | **9** |
| ClinicalTrial.Gov  Date:  *27 March 2026* | **Intervention/treatment:** Esketamine | **323** |
|  | **Other terms:** Anesthesia |  |
| Total | **_** | **2281** |

### **Table S5.** *Outcomes definitions table*

| **Study Name** | **Definition** |
| --- | --- |
| **Emergence delirium** | |
| **Xu 2025** | A PAED score of 10 or higher was interpreted as indicating clinically significant delirium. |
| **Liu 2022** | Postoperative emergence delirium was defined as a PAED score exceeding 10. |
| **Wu 2025** | Emergence delirium was considered present whenever the PAED score reached or exceeded 10. |
| **Zhaoxia 2022** | Emergence agitation was considered present when the agitation score was 3 or 4 on the 4-point scale. |
| **Chen 2023** | An Aono scale score of three or above was regarded as indicative of emergence delirium. |
| **Li 2022** | Emergence delirium was identified as RSS equal to one. |
| **Jiang 2025** | Emergence delirium during recovery was defined as a PAED score ≥12 or a Watch score ≥3. |
| **Li 2025** | Emergence delirium was defined as a PAED score ≥10. |
| **Extubation time** | |
| **Wu 2025** | The duration between anesthesia discontinuation and removal of the endotracheal tube. |
| **Zhaoxia 2022** | The time interval between completion of surgery and tracheal tube removal. |
| **Chen 2023** | The period elapsed between the cessation of anesthetic delivery and tracheal tube removal. |
| **Li 2022** | The precise outcome definition was unclear. |
| **Li 2025** | The time from the end of surgery (discontinuation of anesthetics) until the removal of the tracheal tube. |
| **Pain** | |
| **Liu 2022** | Postoperative pain was assessed using the CHEOPS scale. |
| **Wu 2025** | Postoperative pain was assessed using the FLACC scale at 3, 6, 12, and 24 h. |
| **Zhaoxia 2022** | Postoperative pain was assessed using the modified CHEOPS scale at extubation and PACU discharge. |
| **Chen 2023** | Postoperative pain was assessed using the CHEOPS scale. |
| **Jiang 2025** | Postoperative pain was assessed using the FLACC scale. |
| **Li 2025** | Postoperative pain was assessed using the CHEOPS scale. |
| **Length of PACU stay** | |
| **Consistent across all studies** | The time period from entry to discharge in the PACU. |
| **PONV** | |
| **Consistent across all studies** | Nausea and/or vomiting following surgery. |

### **Table S6:** *Summary findings table with GRADE assessment*

| **Esketamine compared to Placebo for Sevoflurane-Based General Anesthetized Children** | | | | | | |
| --- | --- | --- | --- | --- | --- | --- |
| **Patient or population:** Sevoflurane-Based General Anesthetized Children **Setting:** Patients Undergoing Surgical Procedures in Clinical Trial Settings **Intervention:** Esketamine **Comparison:** Placebo | | | | | | |
| **Outcomes** | **Anticipated absolute effects^*^ (95% CI)** | | **Relative effect (95% CI)** | **№ of participants (studies)** | **Certainty of the evidence (GRADE)** | **Comments** |
|  | **Risk with Placebo** | **Risk with Esketamine** |  |  |  |  |
| **Emergence delirium** | 420 per 1,000 | **147 per 1,000** *(143 to 280)* | **RR 0.35** *(0.26 to 0.48)* | *916 (8 RCTs)* | **⨁⨁⨁◯ Moderate^a,b^** | *Esketamine results in a reduction in Emergence Agitation.* |
| **Pain Score** | - | SMD -**0.58 SD lower** *(-0.79 lower to -0.37 lower)* | - | *(6 RCTs)* | **⨁⨁⨁◯ Moderate^c^** | *Esketamine reduces Pain Score.* |
| **Extubation Time** |  | MD **1.72 min higher** *(0.20 higher to 3.24 higher)* | - | *(5 RCTs)* | **⨁⨁◯◯ Low^c,d^** | *Esketamine probably results in little to no difference in Extubation Time.* |
| **PACU Stay** |  | MD **1.02 min higher** *(-0.73 lower to 2.77 higher)* | - | *716 (7 RCTs)* | **⨁⨁⨁◯ Moderate^c^** | *Esketamine likely does not increase PACU Stay.* |
| **PONV** | 131 per 1,000 | **91 per 1,000** *(84 to 166)* | **RR 0.69** *(0.49 to 0.97)* | *826 (7 RCTs)* | **⨁⨁⨁◯ Moderate^d^** | *Esketamine likely does not reduce PONV.* |
| ***The risk in the intervention group** (and its 95% confidence interval) is based on the assumed risk in the comparison group and the **relative effect** of the intervention (and its 95% CI). **CI:** confidence interval; **MD:** mean difference; **RR:** risk ratio; **SMD:** standardised mean difference | | | | | | |
| **GRADE Working Group grades of evidence** **High certainty:** *We are very confident that the true effect lies close to that of the estimate of the effect.* **Moderate certainty*:*** *We are moderately confident in the effect estimate; the true effect is likely to be close to the estimate, but there is a possibility that it is substantially different.* **Low certainty:** *our confidence in the effect estimate is limited; the true effect may be substantially different from the estimate.* **Very low certainty:** *we have very little confidence in the effect estimate; the true effect is likely to be substantially different from the estimate.* | | | | | | |
| **Explanations:** *a. No downgrade for inconsistency was applied, as the observed heterogeneity was largely explained by meta-regression, primarily due to differences in esketamine doses. b. Egger's Test less than 0.1. c. Significant Heterogeneity less than 0.1. d. High imprecision due to large 95% CI.* | | | | | | |

# **Supplementary Figures**

## **Quality Assessment**

### **Figure S1.** *Traffic light plot*

### **Figure S2.** *Summary plot*

## **Forest Plots**

### **Figure S3.** *Overall emergence delirium*

### **Figure S4.** *Subgroup analysis of the emergence delirium based on route of administration*

### **Figure S5.** *Subgroup analysis of the emergence delirium based on time of administration*

### **Figure S6.** *Subgroup analysis of the emergence delirium based on the type of surgery*

### **Figure S7.** *Subgroup analysis of pain scores based on subanesthetic versus anesthetic dose*

### **Figure S8.** *Overall pain scores*

### **Figure S9.** *Subgroup analysis of pain scores based on specific doses*

### **Figure S10.** *Subgroup analysis of pain scores based on route of administration*

### **Figure S11.** *Subgroup analysis of extubation time based on subanesthetic versus anesthetic dose*

### **Figure S12.** *Overall extubation time*

### **Figure S13.** *Subgroup analysis of extubation time based on specific doses*

### **Figure S14.** *Subgroup analysis of extubation time based on route of administration*

### **Figure S15.** *Subgroup analysis of PACU based on subanesthetic versus anesthetic dose*

### **Figure S16.** *Overall PACU stay*

### **Figure S17.** *Subgroup analysis of PACU based on specific doses*

### **Figure S18.** *Subgroup analysis of PACU based on route of administration*

### **Figure S19.** *Subanesthetic versus anesthetic subgroup analysis for PONV*

### **Figure S20.** *Overall PONV*

### **Figure S21.** *Subgroup analysis of PONV based on specific doses*

### **Figure S22.** *Subgroup analysis of PONV based on route of administration*

## **Sensitivity Analysis**

### **Figure S23.** *Overall leave-one-out sensitivity analysis for the emergence delirium*

### **Figure S24.** *Overall leave-one-out sensitivity analysis for pain scores*

### **Figure S25.** *Overall leave-one-out sensitivity analysis for PACU*

### **Figure S26.** *Overall leave-one-out sensitivity analysis for extubation time*

### **Figure S27.** *Overall leave-one-out sensitivity analysis for PONV*

## **Funnel Plots**

### **Figure S28.** *Overall contour-enhanced funnel plot for the emergence delirium*

### **Figure S29.** *Overall contour-enhanced funnel plot for pain scores*

### **Figure S30.** *Overall contour-enhanced funnel plot for extubation time*

### **Figure S31.** *Overall contour-enhanced funnel plot for PACU stay*

### **Figure S32.** *Overall contour-enhanced funnel plot for PONV*

### **Figure S33.** *Trim and fill funnel plot of emergence delirium.*
